# Draft genome sequences of *Bibersteinia trehalosi* isolates from western Canadian feedlot cattle

**DOI:** 10.1128/mra.00829-26

**Published:** 2026-10-08

**Authors:** Sara Andres-Lasheras, Sujeema Abeysekara, Chloe Brennan, Trevor Alexander, Tim A. McAllister, Rahat Zaheer

**Affiliations:** 1 Lethbridge Research and Development Centre, Agriculture and Agri-Food Canada 6337, Lethbridge, Alberta, Canada; 2 Biological Sciences Department, University of Lethbridge 98636, Lethbridge, Alberta, Canada; University of Manitoba, Winnipeg, Manitoba, Canada

**Keywords:** *Bibersteinia trehalosi*, cattle, bovine respiratory disease, antibiotic resistance

## Abstract

The genomes of 61 *Bibersteinia trehalosi* isolates recovered from respiratory samples of feedlot cattle (Alberta, Canada) were sequenced (Illumina MiSeq). Draft genomes contained genes encoding resistance to up to seven antimicrobial classes relevant to the North American feedlot cattle industry, with integrative and conjugative elements (ICE) associated with resistance genes.

## ANNOUNCEMENT

*Bibersteinia trehalosi* is an opportunistic respiratory pathogen associated with severe outbreaks of bovine respiratory disease (BRD) in feedlot cattle, often resulting in high mortality despite aggressive antimicrobial therapy (1–4). However, its prevalence, contribution to BRD in North America, and genomic determinants of antimicrobial resistance remain poorly understood (5–8).

Respiratory samples were collected from feedlot cattle originating from nine commercial feedlots in Alberta, Canada, between 2017 and 2025 (Animal Use Protocols #231, #1641). Sampled animals included cattle presenting with or that died of BRD (*n* = 30 and *n* = 8, respectively) and clinically healthy cattle (*n* = 6). Deep nasopharyngeal swabs (*n* = 36) and lung tissue samples (*n* = 8) were collected and processed within 24 h and cultured on TSA blood agar with 15 μg/mL bacitracin (9, 10). Suspected *B. trehalosi* colonies were purified and confirmed by PCR or matrix-assisted laser desorption/ionization time-of-flight mass spectrometry (MALDI-TOF MS).

Following the manufacturer’s recommendations, genomic DNA (*n* = 61 isolates) was extracted using the Qiagen Blood & Cell Culture DNA Kit, Genomic-tip 20/G. Libraries were prepared with the Illumina Nextera XT DNA Sample Preparation Kit and sequenced on an Illumina MiSeq platform using MiSeq Reagent Kit V3 (2 × 300 bp paired-end reads), and raw reads were processed on the Galaxy platform (11) using default parameters, unless otherwise noted. Reads were quality-checked using FastQC (v0.12.1) (12), trimmed using Trimmomatic (v0.39 + galaxy2; parameters: headcrop 19, leading 3, trailing 3, sliding window 4:15, minlen 36) (13), assembled with Shovill (v1.4.2 + galaxy1) (14), and annotated using Prokka (v1.14.6 + galaxy1) (15). Assembly quality was evaluated using Quast (v5.3.0 + galaxy1) and BUSCO (v5.8.0 + galaxy2; v5) (16, 17). Antimicrobial resistance genes (ARGs) were identified using Staramr (v0.12.1 + galaxy0) with 80% minimum identity and overlap thresholds (18). ICEs were detected by BLASTn in Geneious v21.0.7 + 6 LTS (100 hits, score 1/−2, gap 2/2, e-value 1e−10, word size 11) using *B. trehalosi* ICEBt190 (CP006956.1) as a reference with 80% minimum identity and coverage thresholds and screening for the core genes *int*, *rel*, *traG*, *traC*, *traD*, *topB1*, *parB*, *parA*, and *dnaB* (19, 20).

Draft genome assemblies averaged 2.38 Mb with a guanine–cytosine (GC) content of 40.8% (Table 1). The most prevalent ARGs included *msr(E)-mph(E)* (82.0%), *tet(H)* (80.3%), and *sul2* (77.0%). All targeted ICE core genes were detected in 10/61 (16.4%) genomes using ICEBerg 3.0 (21). No additional ICE-associated genes were identified in any draft genomes. ARGs, including *msr(E)-mph(E*), *sul2*, and *blaROB-1*, were located within ICE regions.

**TABLE 1:** *Bibersteinia trehalosi* draft genome assembly statistics and quality parameters

Assembly	Average	Median	Min	Max
Coverage	151	107	40	879
#contigs	38	38	23	52
Largest contig (bp)	480,214	473,813	241,379	964,700
Total length (bp)	2,376,078	2,369,344	2,273,109	2,485,185
N50	196,344	186,506	124,498	640,257
GC (%)	40.80	40.81	40.64	40.97
Complete BUSCOs	1,089/1,100	1,089/1,100	1,086/1,100	1,099/1,100

## Data Availability

The raw sequence reads and draft genome assemblies have been deposited in NCBI BioProject PRJNA1482615. This represents the first version of the project. SRA accession numbers SRR39342075–SRR39342135; genome assembly accession numbers JCAIGX000000000–JCAIGZ000000000; JCAIHA000000000–JCAIHZ000000000; JCAIIA000000000–JCAIIZ000000000; and JCAIJA000000000–JCAIJF000000000. Assembly statistics for all genomes are available at https://doi.org/10.5281/zenodo.20800995.
